# Comparative Analysis of Omega-3, Omega-6, and Endocannabinoid Content of Human, Cattle, Goat, and Formula Milk

**DOI:** 10.3390/foods14101786

**Published:** 2025-05-17

**Authors:** Renáta Csatári-Kovács, Tamás Röszer, Éva Csősz

**Affiliations:** 1 Proteomics Core Facility, Department of Biochemistry and Molecular Biology, Faculty of Medicine, University of Debrecen, 4032 Debrecen, Hungary; 2 Metabolomics Research Group, Department of Biochemistry and Molecular Biology, Faculty of Medicine, University of Debrecen, 4032 Debrecen, Hungary; 3 Doctoral School of Molecular Cell and Immune Biology, University of Debrecen, Egyetem tér 1, 4032 Debrecen, Hungary; 4 Department of Pediatrics, Faculty of Medicine, University of Debrecen, 4032 Debrecen, Hungary

**Keywords:** infant nutrition, milk, lipids, breastfeeding

## Abstract

Human milk is the primary source of infant nutrition, although breastfeeding rates are declining today, and human milk is often replaced by animal milk-based infant formula. Infant formula is intended to replicate the composition of human milk, albeit significant differences remain in the physiological responses to breastfeeding and formula feeding in offspring. More research is needed on the composition of human milk and other milk types, especially regarding their lipid content. A comparative analysis of different milk samples was carried out in this study. The amount of omega-3 fatty acids, omega-6 fatty acids, and endocannabinoids was measured in human, cattle, and goat milk as well as in goat milk- and cow milk-based infant formulas using chromatography coupled to mass spectrometry. Significant differences between the human and animal milks were observed in the case of omega-6 fatty acid and endocannabinoid content, with higher omega-6 fatty acid and lower endocannabinoid levels in human milk than in animal milk samples and infant formulas. Goat milk shares the highest similarity to human milk in terms of the analyzed lipid species. However, our results indicate that the levels of the examined bioactive lipid species in human milk failed to be replaced by goat milk- and cow milk-derived infant formulas.

## 1. Introduction

Infant feeding practices determine growth trajectory and body composition in infancy, which have a lasting effect on metabolic health and obesity risk during childhood. Fat catabolism is a major energy source in human newborns, for which the substrate is provided by fat depots and by dietary intake [1,2]. Fat depots begin to develop during the second trimester, with intensive growth in the late third trimester, using maternal ketone bodies and glucose for lipogenesis [1]. It is estimated that as much as 70% of fetal glucose is converted into fat before birth [3,4], and at birth, fat depots contribute to 11–16% of the body weight [2,5].

In the first postnatal months of human infants, there is an extensive fat deposition, especially in the subcutaneous layer, while the visceral fat remains negligible [5,6]. It is estimated that body fat deposition equals 25% of the total energy intake of a newborn [7], and total body fat may reach 30% by 6 months of age [8]. There is an adiposity peak at around the end of the first year of life [9], and body fat should drop to 19–20% by the end of the second year of life and to 14–16% by 10 years of age [8]. An accelerated fat deposition during the first year of life may prevent the drop in body adiposity and may lead to an early adiposity rebound in children, potentially leading to sustained overweight and increased obesity risk as young adults [9,10,11,12].

Human milk is rich in lipids, of which 85–90% are absorbed and metabolized by term infants [4]. The plasma lipid profile of breastfed infants thus reflects the lipid composition of the breast milk [13], and also the maternal adipose tissue and plasma lipids [14,15]. The lipidome of human milk differs considerably from those of infant formula and animal milk, with notable distinctions between ether lipid reflected in the infant plasma lipidome [16,17,18]. Indeed, lipid digestion begins in the buccal cavity in newborns, owing to the presence of lingual lipase in the mouth and lipases of breast milk [19]. Preterm infants have fat malabsorption, although they require increased energy provided by fat.

Recent studies show that a premature transition from breastfeeding to formula feeding—i.e., consuming animal milk instead of human milk—accelerates fat deposition and increases the probability of developing obesity and diabetes later in life [17,20,21,22,23]. Nutritional lipids are not solely energy sources but also function as regulators of energy expenditure by acting as signal molecules [24].

The type of lipids consumed by an infant have an impact on body adiposity. However, still little is known about the differences in lipid profiles of human milk and various animal milk types that are used to manufacture infant formula. A lack or shortening of breastfeeding has adverse effects on adiposity later in life and may also increase the risk of the development of autoimmunity, including type 1 diabetes mellitus during childhood [21,22,23,25]. Favorable effects of breastfeeding have been shown in the establishment of taste preference, cognitive development, neurological development, cardiac fatty acid utilization, blood pressure control, urogenital development, and mucosal immunity [25,26,27,28,29]. Bioactive lipids specific to breast milk may increase energy expenditure during infancy and hence protect from excessive fat deposition [17,30]. Omega-3 fatty acids, omega-6 fatty acids, and endocannabinoid lipid species are present in both human and animal milk, and these lipid species may determine body adiposity and affect inflammation control and neurodevelopment [31,32]. The availability of these lipid species may be different in infant formula and breast milk. These differences may be responsible for the distinct physiological effects of breastfeeding and formula feeding.

Herein, we intended to determine the concentration of omega-3 and omega-6 fatty acids, as well as of endocannabinoid lipid species, in human and animal milk, and in various types of infant formula. We used a pool of breast milk samples collected at our neonatal ward as a representation of human milk. Sample pooling minimizes personal variations in lipid content that may be associated with maternal obesity status, lifestyle, and duration of lactation [33,34]. We used commercially available dairy milk and infant formula as a comparison. Our analysis was restricted to infant formulas that are used in our neonate ward.

## 2. Materials and Methods

### 2.1. Chemicals and Reagents

Lipid standards arachidonic acid (AA), alpha-linolenic acid (ALA), docosahexaenoic acid (DHA), eicosapentaenoic acid (EPA), linoleic acid (LA), oleoylethanolamide (OEA), palmitoylethanolamide (PEA), and formic acid were obtained from Sigma-Aldrich (St. Louis, MO, USA). 2-Arachidonylglycerol (2-AG) was purchased from Avantii Research (Alabaster, AL, USA). Stable isotope-labeled (SIL) palmitoyl ethanolamide-d_4_ (PEA-d_4_) was purchased from Cayman Chemical (Ann Arbor, MI, USA). HPLC-grade methanol, acetonitrile, and LC-MS-grade water were obtained from VWR Ltd. (Radnor, PA, USA).

### 2.2. Milk Samples

Human breast milk, goat milk, cow milk, goat milk-based, and cow milk-based infant formula were analyzed in this study. Human breast milk samples were donated by nursing mothers at the neonatology ward of the Department of Pediatrics, University of Debrecen, and were pooled and used for analysis without the possibility of identifying personal information of the donors. Breast milk samples were obtained with breast pumps. In general, donors were at the age of 25–28 years, without seropositivity for SARS-CoV-2, maternal diabetes, asthma, or mastitis. The goat milk-based infant formula (Holle, Riehen, Switzerland), the cow milk-based infant formula (Nestlé, Vevey, Switzerland), the raw cow milk (local market, Debrecen, Hungary), and the 1.5% fat goat milk (Andechser Natur, Andechs, Germany) were commercially available products. The commercially available dairy milk samples were pools of various batches. These samples were analyzed freshly, while the infant formulas were reconstituted with warm water according to the guidelines provided on the packaging and used immediately.

### 2.3. Preparation of Standards and Samples

We used 100 µL of each sample and placed it in 2 mL microcentrifuge tubes. The lipid components were extracted with 1 mL methanol. The samples were vortexed for 10 s and put in the ice for 1 min. This was followed by a centrifugation step at 16,900× *g*, 4 °C for 10 min (Eppendorf Centrifuge 5418 R, Hamburg, Germany). After centrifugation, 950 µL of the supernatants were removed and placed in new 2 mL microcentrifuge tubes and then dried in a vacuum concentrator (Eppendorf Concentrator 5301, Hamburg, Germany) for 20 min. Lipid stock solution was prepared in methanol containing 10 µg/mL of 2-AG, AA, DHA, EPA, OEA, and PEA; 62.5 µg/mL ALA; and 187.5 µg/mL LA. The calibration standards were prepared from the stock solution through serial dilutions. An 11-point calibration curve was prepared containing 0.0001, 0.0005, 0.001, 0.005, 0.01, 0.025, 0.05, 0.1, 0.5, 1, and 2.5 µg/mL of each analyte except ALA and LA. In the case of LA, the concentrations were 0.0075, 0.0375, 0.075, 0.375, 0.75, 1.875, 3.75, 7.5, 37.5, 75, and 187.5 µg/mL, while for ALA the concentrations were 0.0025, 0.0125, 0.025, 0.125, 0.25, 0.625, 1.25, 2.5, 12.5, 25, and 62.5 µg/mL. The stable isotope-labeled internal standard was prepared with PEA-d_4_ in a 1 µg/mL final concentration. A total of 98 µL of milk sample or calibration standard and 2 µL SIL standard were pipetted into brown glass HPLC vials with a glass insert (Waters, Milford, MA, USA). For analyses, 5 µL of milk samples or calibration standard solution was injected in duplicate.

### 2.4. Determination of Lipid Concentrations with UHPLC–MS

The concentration of selected lipids in milk and infant formula samples was determined using the Acquity H-Class UPLC system (Waters, Milford, MA, USA) coupled to a 5500 QTRAP mass spectrometer (Sciex, Framingham, MA, USA). Liquid chromatographic separation was performed on an AccQ-tag Ultra C18 column (1.7 µm; 2.1 × 100 mm, Waters, Milford, MA, USA) guarded by an Acquity in-line filter (0.2 µm; 2.1 mm, Waters, Milford, MA, USA). An in-house-developed 15 min-long gradient was used with a 0.4 mL/min flow rate and a 40 °C column temperature. Solvent A was LC-MS-grade water:acetonitrile (80:20, vol/vol) with 0.1% formic acid, and solvent B was 100% acetonitrile with 0.1% formic acid. Table 1 contains the elution profile of the UPLC separation.

The SRM-based targeted mass spectrometry analyses were carried out on a 5500QTRAP mass spectrometer controlled by Analyst software (version 1.6.3., Sciex, Framingham, MA, USA). We used electrospray ionization with a 4500 V spray voltage, and SRM spectra were recorded in positive or negative ion mode. The ion source gas 1 and 2 were 30 psi and 50 psi, respectively; the curtain gas was 30 psi; and the source temperature was 500 °C. The detailed parameters of the SRM experiment are presented in Table 2.

The SRM spectra were analyzed with Skyline software (version 24.1.0.199), and the concentrations were calculated using the recorded calibration curve. For statistical analysis of the data, we used one-way ANOVA with Dunnet’s post hoc test, using human breast milk as a reference group. Statistical significance is indicated in the graphs as follows: * *p* < 0.05, ** *p* < 0.01, *** *p* < 0.001, comparing the values to the corresponding values measured in breast milk. We used GraphPad Prism (version 8.0.1 for Windows, GraphPad Software v5, San Diego, CA, USA, www.graphpad.com, accessed on 15 May 2025) statistical software for statistical analysis.

## 3. Results

**Figure 1:**
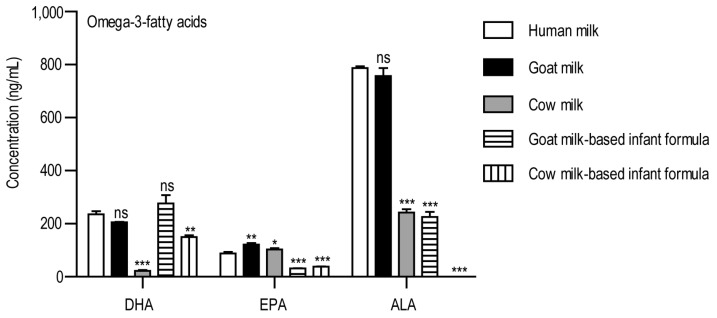
The concentrations of omega-3 PUFAs in the examined milk samples. The y-axis shows the concentrations in ng/mL, while the examined analytes are shown on the x-axis. Abbreviations: DHA: docosahexaenoic acid; EPA: eicosapentaenoic acid; ALA: alpha-linolenic acid. ns: non-significant, * *p* < 0.05, ** *p* < 0.01, *** *p* < 0.001, one-way ANOVA with Dunnett’s post hoc test, using human milk as reference.

In this study, the concentration of selected lipids was determined in different types of milk using liquid chromatography coupled to a mass spectrometry. A total of five milk types were analyzed, and the amount of omega-3 and omega-6 polyunsaturated fatty acids (PUFA) and eicosanoids was examined. Raw mass spectra data are available in the Panorama public data repository. Among the omega-3 fatty acids, we determined the concentration of ALA, EPA, and DHA (Figure 1).

EPA and DHA were present in all of the examined milk types, but we could not detect ALA in the cow milk-based infant formula. The goat milk-based infant formula contained the highest concentration of DHA, followed by human milk, goat milk, and cow milk-based infant formula. The lowest amount of DHA was detected in cow milk. The goat milk contained the highest concentration of EPA, followed by cow milk and human milk. The amount of EPA was almost equal in the goat milk-based and cow milk-based infant formulas. Regarding ALA, the human milk and goat milk contained the highest concentrations of ALA, followed by cow milk-based and goat milk-based infant formulas, which had ALA almost in equal amounts.

**Figure 2:**
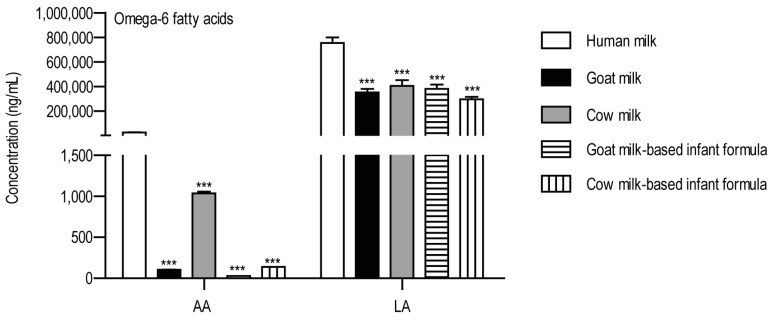
The concentrations of omega-6 fatty acids in the examined milk samples. The y-axis shows the concentrations in ng/mL. The studied analytes are shown on the x-axis. Abbreviations: AA: arachidonic acid; LA: linoleic acid. *** *p* < 0.001, one-way ANOVA with Dunnett’s post hoc test, using human milk as reference.

Among the omega-6 fatty acids, the concentrations of AA and LA were examined (Figure 2).

Both AA and LA were present in all the samples. AA was most abundant in breast milk, followed by cow milk. The amount of AA was very low in the other samples. Similar to AA, the amount of LA was the highest in the human milk. In the other types of samples, the concentration of LA was almost equal and lower than in human milk.

**Figure 3:**
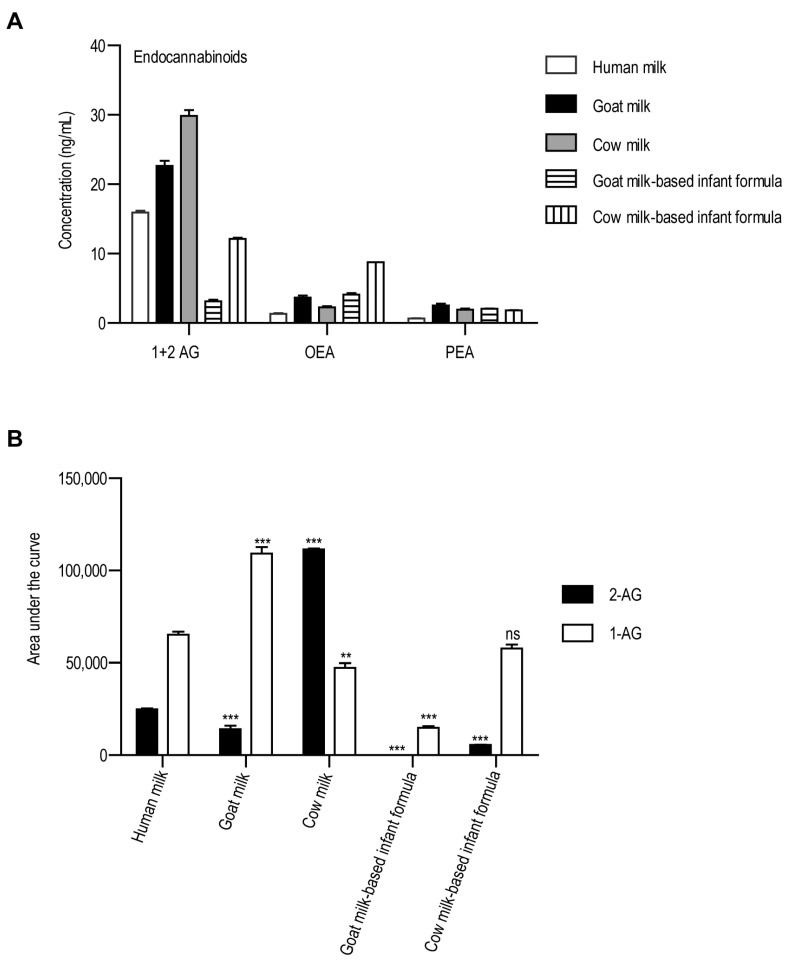
The concentrations of endocannabinoids in the examined milk samples. The y-axis shows the amount of analytes, while the studied analytes are shown on the x-axis. (**A**) Concentration of 1-AG + 2-AG, OEA, and PEA. (**B**) The relative quantification of 2-AG and 1-AG based on the area under the curve. Abbreviations: 2-AG: 2-arachidonylglycerol; OEA: oleoylethanolamide; PEA: palmitoylethanolamide. ns: non-significant, ** *p* < 0.01, *** *p* < 0.001, one-way ANOVA with Dunnett’s post hoc test, using human milk as reference.

The concentrations of endocannabinoids 2-AG, 1-AG, OEA, and PEA were examined (Figure 3A). All analytes could be detected in all milk samples examined. 2-AG is the active form acting on CB1 receptors and can spontaneously isomerize to inactive 1-AG [35]. This spontaneous isomerization occurred in the calibration solutions as well, so we could not obtain accurate information on the amount of 2-AG.

Using the calibration curve, we could only obtain information on the 1-AG + 2-AG together, and we used the area under the curve to obtain the relative amount of 2-AG and 1-AG (Figure 3B). The amount of 2-AG was the highest in cow milk, and we could not detect it in the goat milk-based infant formula. The amount of 1-AG was almost two times higher than that of 2-AG in all samples except the cow milk, where the amount of 2-AG was almost double that of 1-AG.

The amount of OEA and PEA was the lowest in human milk, and the amount of OEA was the highest in the cow milk-based infant formula, followed by goat milk and the other samples.

## 4. Discussion

Exclusive breastfeeding for the first six months of life is the current recommendation in infant care; however, there are various factors that may impede or shorten breastfeeding [36]. In such cases, breast milk is often substituted with infant formula [37]. The composition of infant formulas is intended to resemble human milk as much as possible. Depending on the cow or goat milk they use as a base, manufacturers modify the composition of their products, fortifying them with specific lipids or carbohydrates and/or lysing the milk proteins to achieve a better absorption and lower immunogenicity [38,39].

Regulatory agencies have recommendations regarding the amount of bioactive lipids in infant formulas. According to the Codex Alimentarius, there is a minimum limit for LA and a minimum and maximum for ALA. If DHA is added to infant formula, AA also has to be present in at least the same concentration as DHA. The level of EPA should not exceed the DHA level (Codex Alimentarius).

**Figure 4:**
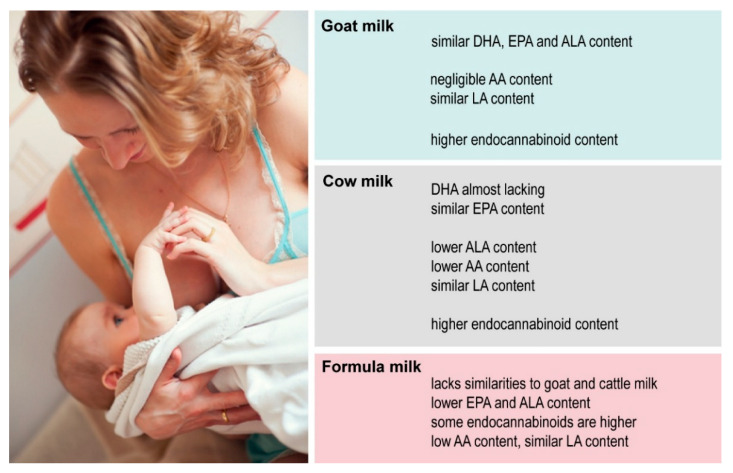
Similarities and differences in the lipid composition of human milk to goat milk, cow milk, and infant formula. The scheme highlights the key features of goat milk, cow milk, and infant formula that are similar to or different from human milk. Image courtesy of Dreamstime Stock Photos & Images, ID 49203333.

There are studies that examine the lipid content of human milk, infant formula, and animal milk [16,40,41], but information about the comparison of all these type of milks regarding the bioactive lipid content is scarce. In our study, the comparative analysis of human, goat, and cow milk as well as goat milk-based and cow milk-based infant formulas regarding the selected bioactive lipids showed the distinct composition of these milk types regarding the eight examined lipid species belonging to omega-3 and omega-6 fatty acids, as well as endocannabinoids. Significant differences between the human and animal milks were observed in the case of omega-6 fatty acids and endocannabinoids, with higher omega-6 fatty acids and lower 2-AG, PEA, and OEA levels in human milk compared to animal milk samples and infant formulas (Figure 4).

Omega-3 and -6 PUFAs are important constituents of human nutrition [32,42,43,44] and, along with endocannabinoids, have various biological effects beyond serving as energy sources. They have a role in inflammation, mitochondrial functioning, and fat metabolism and have a cardioprotective effect, and some of them, such as AA, EPA, and DHA, have protective effects in type 2 diabetes mellitus [32,42,43]. Their functions have mostly been examined in adults; the information on their effect in newborns and infants is scarce. Considering the profound effect of PUFAs and endocannabinoids on fat metabolism and the high dependency of the plasma lipid profile of infants on the lipid composition of food, ideally breast milk [45], we were eager to examine the amount of some PUFAs and endocannabinoids in milk and infant formulas.

Omega-3 PUFAs such as ALA, EPA, and DHA in adults can help prevent inflammation and platelet aggregation, reduce the triglyceride level in the liver [44,46], and, at the cellular level, increase mitochondrial biogenesis and fatty acid oxidation [47]. ALA is an essential fatty acid; it cannot be synthesized by the human body and should be taken up with food [48]. It decreases the risk of cardiovascular diseases (CVDs) and has a cholesterol-lowering effect [49].

EPA and DHA have anti-inflammatory, cardioprotective, and hypotriglyceridemic properties [35,50], with DHA also playing a role in brain health [51]. Regarding their role in adipogenesis, it was shown that ALA and EPA inhibit adipogenesis when introduced at the differentiation stage of 3T3-L1 cell cultures [52]. Human milk, along with goat milk, can be considered a good source of ALA, EPA, and DHA, while the levels of ALA and EPA were much lower in infant formulas, with ALA missing from the cow milk-based formula. Deprivation of omega-3 PUFAs during pregnancy impairs visual acuity in Rhesus monkeys [53] and may delay neurodevelopment in human newborns, causing lower language development and visual acuity [54]; however, their necessity in human development is still unclear [55]. It is still to be defined whether there is a consistent benefit of omega-3 PUFA supplementation during pregnancy and lactation on neurodevelopment and visual acuity in term infants [32]. Similarly, dietary supplementation with omega-3 PUFAs and reducing AA intake during pregnancy and lactation do not affect fat mass in offspring [56]. Newborns deprived of breast milk feeding are at increased risk of necrotizing enterocolitis (NEC), a severe and potentially life-threatening inflammatory condition [57]. In animal studies, dietary supplementation with omega-3 PUFAs during lactation reduces the severity of NEC [58,59,60]. However, DHA supplementation may increase the risk of NEC in human newborns [61].

The omega-6 fatty acid LA is a precursor of other omega-6 fatty acids, such as gamma-linolenic acid (GLA) and AA [11]. AA is an important polyunsaturated fatty acid and is the component of cell membranes, can modulate the cell membrane fluidity, and can be the precursor of prostaglandins and leukotrienes [46]. High intake of omega-6 PUFAs may be associated with weight gain and obesity, although the evidence is not conclusive [47]. They may affect appetite regulation and fat metabolism, potentially leading to increased caloric intake and adiposity [62]. However, reduced levels can have immediate effects; it has been shown that in term infants, an inadequate concentration of LA leads to suboptimal growth and skin problems [16]. According to our data, human milk was the best source of AA and LA; however, the level of LA was relatively high in all examined milk types. A high AA/EPA + DHA ratio during the first 4 months of life is significantly associated with infant adiposity [31]. Accordingly, a low dietary omega-6/omega-3 PUFA ratio during early postnatal life increases fatty acid oxidation and favors the development of thermogenesis and lipid catabolizing adipocytes in mouse models [63]. However, reducing AA intake during pregnancy and lactation does not affect fat mass in human infants [56].

It is recommended that infant formulas contain balanced LA, ALA, AA, and DHA levels. In the EU and in China, DHA should be added to infant formula in amounts that resemble the DHA level in human milk [16].

Endocannabinoids can help regulate physiological and behavioral responses to stress [64]. Breast milk endocannabinoids are necessary for initiating suckling movements in newborns [65], and by affecting neuronal development, they may affect food preference in the long term [33]. 2-AG is a monoacylglycerol acting on the cannabinoid CB1 receptor, and in addition to the initiation of suckling movements after birth, it has an important role in lipid metabolism by stimulating lipogenesis [66,67]. For instance, a lack of adipocyte CB1 receptor protects mice from obesity and increases sympathetic tone in adipose tissue, favoring lipolysis [68]. CB1 receptors are necessary for axon development during early neurodevelopment and for the postnatal development of appetite control [69,70]. Systemic loss of CB1 prevents the initiation of suckling in newborn mice [71]. Recent evidence links human obesity to the activation of the endocannabinoid system and demonstrates the role of increased endocannabinoid levels in both central and peripheral tissues, along with CB1 receptor up-regulation in obesity [70]. The active 2-AG can spontaneously isomerize to inactive 1-AG [35], damping in this way the effect of 2-AG and posing a pre-analytical challenge in the examination of this compound [72]. According to our data, cow milk was the best source of 2-AG, while in human and goat milk the amount of 1-AG was dominant, but 2-AG was also present. The analyzed infant formulas contained negligible amounts of 2-AG and only some 1-AG. It should be mentioned that the results of our measurements reflect the conditions present in the sample, with no information available either on the physiological state or ton he dynamics of 2-AG–1-AG transformation in the sample.

PEA is an endocannabinoid-like lipid mediator that has anti-inflammatory, antimicrobial, immunomodulatory, and neuroprotective effects [73]. OEA is an endogenous lipid mediator that is synthesized from membrane glycerophospholipids. It stimulates lipolysis and fatty acid oxidation by activating PPARα and activates signaling pathways responsible for reducing food intake [74,75]. Our comparative analysis revealed that the level of OEA and PEA was the lowest in human milk. A very high amount of OEA, almost seven times higher than that of human milk, was observed in the cow milk-based infant formula. Excessive stimulation of the endocannabinoid system in the limbic system and the hypothalamus may account for increased food intake and positive energy balance [65,70]. In a study involving adults with diet modification, it was observed that the concentrations of 2-AG, PEA, and OEA were positively associated with body fat percentage [76]. To our knowledge, corresponding studies are not available in infants. We can only speculate that formula feeding may create a positive energy balance and increase obesity risk later in life [21], as it increases lipogenesis [17] and may shift food preferences towards high energy intake in the long term [77]. It is plausible that excessive endocannabinoid intake by formula feeding may be a contributor to these effects, although this should be defined by future research. 

A limitation of our study is that it uses only one cow milk-based and one goat milk-based infant formula product, and also it cannot answer questions regarding the function of the analyzed lipid species in infants. It can only give information on the differential composition of different milk and milk-based formulas, with the possibility of using them for infant feeding. Our analysis suggests that breast milk, dairy milk, and milk-based formula have their own unique lipid composition, which warrants caution when breastfeeding is limited.

Taking human milk as the gold standard for feeding newborns and infants, goat milk has the most similar content considering the examined lipid species. It has been shown that only goat milk-based formula provides alkyl-glycerols, which are breast milk-specific lipid species with a protective role against obesity [17].

## 5. Conclusions

The results of our comparative analysis indicate the superiority of human milk over goat and cow milk or infant formulas using these types of milks. Our results can provide a starting point for further research regarding balancing the bioactive lipid content of infant formulas to resemble as much as possible the composition of human milk.

## Figures and Tables

**Table 1:** Gradient applied during the UPLC separation. The time, the flow rate, and the percentage of solvent A and B are shown.

Time (min)	Flow Rate (mL/min)	Solvent A (%)	Solvent B (%)
0.00	0.40	75.00	25.00
3.00	0.40	60.00	40.00
4.00	0.40	40.00	60.00
5.00	0.40	30.00	70.00
6.00	0.40	14.00	86.00
10.00	0.40	11.00	89.00
10.50	0.40	0.00	100.00
12.00	0.40	0.00	100.00
13.50	0.40	75.00	25.00
15.00	0.40	75.00	25.00

**Table 2:** SRM parameters used during analysis. The detection mode and SRM transition parameters along with the retention time window, declustering potential, and collision energy are presented for each analyte. Q1 m/z: parent ion m/z, Q3 m/z: fragment ion m/z, tR window: retention time window in minutes, DP: declustering potential in eV, CE: collision energy in eV.

Compound	Detection Mode	Q1 (m/z)	Q3 (m/z)	t_R_ Window (Min)	DP (eV)	CE (eV)
2-AG	Positive	379.00	305.00	5.7–7.5	100	30
2-AG	Positive	379.00	287.00	5.7–7.5	100	30
2-AG	Positive	379.00	269.00	5.7–7.5	100	30
2-AG	Positive	379.00	259.00	5.7–7.5	100	30
OEA	Positive	326.00	265.00	5.9–7.7	100	30
OEA	Positive	326.00	247.00	5.9–7.7	100	30
OEA	Positive	326.00	135.00	5.9–7.7	100	30
OEA	Positive	326.00	121.00	5.9–7.7	100	30
OEA	Positive	326.00	62.00	5.9–7.7	100	30
PEA	Positive	300.00	283.00	5.7–7.5	100	30
PEA	Positive	300.00	123.00	5.7–7.5	100	30
PEA	Positive	300.00	109.00	5.7–7.5	100	30
PEA	Positive	300.00	95.00	5.7–7.5	100	30
PEA	Positive	300.00	62.00	5.7–7.5	100	30
PEA-d_4_	Positive	304.00	287.00	5.7–7.5	100	30
PEA-d_4_	Positive	304.00	97.00	5.7–7.5	100	30
PEA-d_4_	Positive	304.00	62.00	5.7–7.5	100	30
DHA	Negative	327.00	283.00	6.0–7.8	−220	−20
DHA	Negative	327.00	281.00	6.0–7.8	−220	−20
DHA	Negative	327.00	229.00	6.0–7.8	−220	−20
EPA	Negative	301.00	257.00	5.7–7.5	−220	−25
EPA	Negative	301.00	255.00	5.7–7.5	−220	−25
EPA	Negative	301.00	203.00	5.7–7.5	−220	−25
AA	Negative	303.00	259.00	6.2–8.0	−220	−30
AA	Negative	303.00	231.00	6.2–8.0	−220	−30
AA	Negative	303.00	205.00	6.2–8.0	−220	−30
AA	Negative	303.00	177.00	6.2–8.0	−220	−30
LA	Negative	279.00	261.00	6.3–8.1	−220	−35
LA	Negative	279.00	127.00	6.3–8.1	−220	−35
LA	Negative	279.00	71.00	6.3–8.1	−220	−35
ALA	Negative	277.00	233.00	5.8–7.6	−220	−38
ALA	Negative	277.00	127.00	5.8–7.6	−220	−38
ALA	Negative	277.00	71.00	5.8–7.6	−220	−38

## Data Availability

The original data presented in the study are openly available in Panorama: https://panoramaweb.org/University%20of%20Debrecen/Milk_project/project-begin.view.

## Abbreviations

The following abbreviations are used in this manuscript:

AA	Arachidonic acid
ALA	Alpha-linolenic acid
DHA	Docosahexaenoic acid
EPA	Eicosapentaenoic acid
LA	Linoleic acid
OEA	Oleoylethanolamide
PEA	Palmitoylethanolamide
PUFA	Polyunsaturated fatty acid
